# NGF Modulates trkA^NGFR^/p75^NTR^ in αSMA-Expressing Conjunctival Fibroblasts from Human Ocular Cicatricial Pemphigoid (OCP)

**DOI:** 10.1371/journal.pone.0142737

**Published:** 2015-11-16

**Authors:** Alessandra Micera, Barbara Stampachiacchiere, Antonio Di Zazzo, Roberto Sgrulletta, Magdalena Cortes, Eduardo Maria Normando, Alessandro Lambiase, Stefano Bonini

**Affiliations:** 1 IRCCS-G.B. Bietti Foundation, Rome, Italy; 2 Department of Ophthalmology, University Campus Bio-Medico, Rome, Italy; 3 Glaucoma & Retinal Degeneration Research Group, Visual Neurosciences, UCL Institute of Ophthalmology, 11–43 Bath Street, London, EC1V 9EL, United Kingdom; 4 The Western Eye Hospital, Imperial College Healthcare Trust, Marylebone Road, London, NW1 5QH, United Kingdom; 5 Ophthalmology, Dept. Organi di senso, University of Rome “Sapienza”, Rome, Italy; University of Edinburgh, UNITED KINGDOM

## Abstract

**Objective:**

In a previous study, we reported the upregulation of Nerve Growth Factor (NGF) and trkA^NGFR^ expression in Ocular Cicatricial Pemphigoid (OCP), an inflammatory and remodeling eye disease. Herein, we hypothesize a potential NGF-driven mechanism on fibroblasts (FBs) during OCP remodeling events. To verify, human derived OCP-FBs were isolated and characterized either at baseline or after NGF exposure.

**Materials and Methods:**

Conjunctival biopsies were obtained from 7 patients having OCP and 6 control subjects (cataract surgery). Both conjunctivas and primary FB cultures were characterised for αSMA, NGF and trkA^NGFR^/p75^NTR^ expression. Subcultures were exposed to NGF and evaluated for αSMA, NGF, trkA^NGFR^/p75^NTR^ expression as well as TGFβ1/IL4 release. For analysis, *early and advanced* subgroups were defined according to clinical parameters.

**Results:**

OCP-conjunctivas showed αSMA-expressing FBs and high NGF levels. A*dvanced* OCP-FBs showed higher αSMA expression associated with higher p75^NTR^ and lower trkA^NGFR^ expression, as compared to *early* counterparts. αSMA expression was in keeping with disease severity and correlated to p75^NTR^. NGF exposure did not affect trkA^NGFR^ levels in *early* OCP-FBs while decreased both αSMA/p75^NTR^ expression and TGFβ1/IL4 release. These effects were not observed in *advanced* OCP-FBs.

**Conclusions:**

Taken together, these data are suggestive for a NGF/p75^NTR^ task in the potential modulation of OCP fibrosis and encourages further studies to fully understand the underlying mechanism occurring in fibrosis. NGF/p75^NTR^ might be viewed as a potential therapeutic target.

## Introduction

The Ocular Cicatricial Pemphigoid (OCP) is an immune-mediated chronic inflammatory disease of the eye, characterized by chronic-recurrent conjunctival inflammation, progressive sub-epithelial fibrosis and tissue remodeling [1–3]. Inflammatory infiltrates and activated Fibroblasts (FBs) contribute actively to the uncontrolled extracellular matrix (ECM) deposition (remodeling process), leading to structural and functional changes (keratinization and blindness) [3,4]. Several pro-inflammatory/fibrogenic cytokines and growth factors, including Transforming Growth Factor β1 (TGFβ1) and Interleukin 4 (IL4), have shown the ability to modulate the survival of activated FBs and their collagen deposition [2,5–7].

An involvement of Nerve Growth Factor (NGF) pathway in OCP has been previously reported by our group: an increased trkA^NGFR^ immunoreactivity has been observed in OCP conjunctival stroma and a consistent NGF release has been quantified in OCP tears [8,9]. The effect of NGF in tissue remodelling and fibroblast activity is actually controversial: NGF might exert pro/anti-inflammatory effects or profibrogenic activity, acting as a “modulator” of the local immune/inflammatory response, in a receptor expression dependent manner [10–16]. In the last decade, the NGF modulatory effect on Fibroblasts (FBs) and their activated myofibroblast counterpart (myoFBs) has been prospected in view of the surface trkA^NGFR^/p75^NTR^ rate expression and the NGF ability to trigger apoptosis in FBs from different tissues as well as TGFβ1-induced myoFBs [17–21].

To address the question as whether NGF might modulate OCP-fibrosis, activated FBs and NGF immunoreactivity were verified both in tissues and cultures. Next, the study was extended to the *in vitro* characterization of OCP-FBs and the potential NGF influence on OCP-FB phenotype by monitoring αSMA, trkA^NGFR^/p75^NTR^ and TGFβ1/IL4 in NGF-exposed OCP sub-cultures.

## Materials and Methods

### Ethics Statement

The study followed the guidelines of the Declaration of Helsinki for research involving human subjects and was approved by the intramural Ethical committee (UCBM). Informed written consent was signed by each patient adhering to the study.

### Patients and conjunctival specimens

Conjunctival biopsies were obtained from 7 patients with clinical and histological (Hematoxylin & Eosin, HE; Bio-Optica, Milan, Italy) diagnosis of OCP (2M/5F; mean age, SD, range 55–88 years) and from 6 healthy age matched patients (control group), during routine cataract surgery (5M/1F; mean age, SD, range 59–81 years).

Two fragments were produced from each biopsy: one conjunctival fragment was included in paraffin and sectioned to provide 5μm-sections for light/confocal microscopy, while the other fragment was used to achieve primary culture of conjunctival OCP FBs.

OCP specimens were classified according to the stage of the disease [22,23] and grouped as follows: early group comprising 3 patients (stages I or II; Foster) and advanced group including 4 patients (stages III or IV). The immunofluorescent analysis was performed for identifying the presence of a linear immunoglobulin deposition alongside the Basament Membrane Zone (BMZ), according to the specific immunoreactivity (FC-coupled IgGAM antibodies; OBT0119F; Oxford Biotech., Oxford, UK), univocally present in OCP positive sections. Basal histology included Giemsa (48900; Fluka, Milan, Italy), Haematoxylin and Eosin (HE; 05-M06014/05-M10002;Bio-Optica; Milan, Italy) as well as the Periodic Acid Schiff (PAS; 04-130802/05-M06002; Bio-Optica; Milan, Italy) stainings.

All sterile tissue culture plastic-ware and reagents were from NUNC (Roskilde, Denmark) and Serva (Heidelberg, Germany). Culture media and supplements were from Euroclone (Milan, Italy). Ultrapure/RNAse free water was provided by Direct-Q5 Apparatus (Millipore, Vimodrone, Milan, Italy).

### Explants, FB subcultures and NGF studies

Conjunctival fragments were put as explants in 24-well plates and left to attach for 10min before adding DMEM supplemented with 10% heat-inactivated Fetal Bovine Serum (FBS), 1mM sodium Pyruvate, 2mM glutamine, 100U/mL penicillin and 100μg/mL streptomycin (37°C, 5% CO_2_ in air) [17,18]. Outgrowing FBs were quickly harvested (0.2% trypsin-0.025% EDTA; HyQ trypsin; HyClone, Waltham, MA) and directly used or sub-cultured in T-21cm^2^/T-75cm^2^ flasks (3^rd^-5^th^ passage) for NGF exposure. The cell culture purity was estimated by the CK19 exclusion test (anti-cytokeratin 19 conjunctival epithelial marker, 1/100; Dako, Hamburg, Germany).

For stimulation studies, serum starved confluent monolayers were exposed to increasing NGF concentrations (0 to 100ng/mL β-NGF Grade I; Alomone Labs Ltd, Jerusalem, Israel) or 1ng/mL TGFβ1 (positive control; R&D system, Minneapolis, MN) performed in 0.5% FBS-DMEM for 24hrs.

Conditioned media were collected for ELISA/Western Blot analysis, while monolayers were directly processed for confocal analysis or treated with trypsin-EDTA solution to obtain single cells for molecular (10^5^ cells) / biochemical (10^6^ cells) analysis.

### Confocal Analysis

Conjunctival sections and monolayers were subjected to fluorescent immunostaining. Briefly, de-waxed sections and confluent monolayers on round coverslips (Mierfield, USA) were washed in Hank’s Balanced Sodium Salt (HBSS), fixed in 2% buffered ρ-Formaldehyde (PFA), equilibrated in PBS [10mM phosphate buffer and 137mM NaCl; pH 7.5], briefly permeabilized with 0.5% Triton X100 in PBS (TX-PBS) and probed with the specific antibodies, either alone or in combination: mouse anti-human αSMA antibodies (1/60; Novocastra, Newcastle, UK), goat anti-human NGF antibody (sc-549; 1/100); rabbit anti-human trkA^NGFR^ (sc-118; 1/150) and goat anti-human p75^NTR^ (sc-6188; 1/75) antibodies (all from Santa Cruz Biotech., Santa Cruz, CA). The specific binding was detected by using Cy2/Cy3-conjugated specie-specific secondary antibodies, depending on the specific staining (1/500-1/700; Jackson ImmunoResearch Labs., Europe Ltd, Suffolk, UK). Nuclei were counterstained with Propidium Iodide (5μg/mL; ICN, Milan, Italy). Acquisitions were carried out using the E2000U confocal microscope equipped with C1 software (Nikon, Tokyo, Japan). Control sections were stained in parallel (control irrelevant IgGs; Vector Laboratories, Inc. Burlingame, CA) and used for the channel-series acquisitions (Nikon).

### Flow cytometry

Single-cells were washed in HBSS containing Ca^++^/Mg^++^ and fixed in 3.7% buffered PFA. After washing, cells were either directly immunostained or further incubated in 70% methanol in PBS (20°C, 24hrs). For staining, cells were equilibrated in FACS buffer [0.1% saponin and 0.1% NaN_3_ in PBS; pH 7.5] and incubated with the above reported primary antibodies diluted in 0.1% BSA-FACS buffer. The specific binding was detected with Cy2/PE-conjugated specie-specific secondary antibodies (1/600-1/700; Jackson). Cells (10^4^ events) were analysed using the MACSQuant flow cytometer and cell plots were arranged using the manufacturers’ provided software (Miltenyi Biotech., Gladbach, Germany). Changes in Mean Fluorescence Intensity (MFI) were calculated as follows: ΔMFI = [(MFI_specific_/ MFI_not-specific_] and ΔMFI values ≥1 were used for statistical analysis.

### Relative Real-Time PCR analysis

Total RNA was extracted from confluent monolayers using the Puregene RNA purification kit (Gentra Systems, Minnesota, USA). Total RNA samples were spectrophotometrically analysed (ND-1000; NanoDrop, Wilmington, DE; λ_260_/λ_280_>1.8). Total RNA (3 μg) samples were reverse transcribed to a final volume of 21μlL, using 50 pM oligo dT21-primer, 1 mM dNTP mix and 200 U reverse transcriptase (IMPROM; Promega, Milan, Italy) in a programmable PTC100 thermocycler (MJ Research, Watertown, MA) and 3μL were run for amplification with the specific target/referring primers in an Opticon2 MJ thermocycler (MJ Research), according to the manufacturers’ instructions (Table 1). PCR experiments were carried out in a final volume of 20μL containing 3μL cDNA for target genes (or 1μL/3μL for GAPDH/H3 reference genes) and 17μL of master mix [10μL SYBR Green PCR Mix (Applied Biosystems, Foster City, CA), 0.5μL of each primer (10 pM; MWG, Biotech, Ebersberg, Germany) and milliQ-water]. Amplicons were verified for their specificity according to the Southern blotting analysis. Negative controls (without template or with total RNA) were carried out for each run, to rule out any genome contamination. Single threshold cycle values (Cts) were run in the REST 384–2006 software [24] to get increase or decrease difference in target gene expression, with respect to reference genes and compared to controls. Data are gene expression ratio provided in log2 scale.

**Table 1:** Primers and amplification program used in the study.

Primer	Sequence	bp	Ta*	Accession number
**NGF**	for 5'-CTG GCC ACA CTG AGG TGC AT-3'	120	55°C, 30sec	**BC011123**
	rev 5’-TCC TGC AGG GAC ATT GCT CTC-3’			
**trkA** ^**NGFR**^	for 5'-CAT CGT GAA GAG TGG TCT CCG-3'	102	57°C, 25sec	**M23102**
	rev 5'-GAG AGA GAC TCC AGA GCG TTG AA-3'			
**p75** ^**NTR**^	for 5'-CCT ACG GCT ACT ACC AGG ATG AG-3'	147	57°C, 25sec	**AF187064**
	rev 5’-TGG CCT CGT CGG AAT ACG-3’			
αSMA	for 5'-GAA GGA GAT CAC GGC CCT A-3'	125	55°C, 30sec	**BC017554**
	rev 5’-ACA TCT GCT GGA AGG TGG AC-3’			
**TGFβ1**	for 5'-TCC TGG CGA TAC CTC AGC AA-3'	110	57°C, 25sec	**BC017288**
	rev 5’-GCC CTC AAT TTC CCC TCC AC-3’			
**GAPDH**	for 5'-CCT GAC CTG CCG TCT AGA AA-3'	111	55°C, 30sec	**BC013310**
	rev 5’-ACC TGG TGC TCA GTG TAG CC-3’			
**H3**	for 5’-GCT TCG AGA GAT TCG TCG TT-3’	113	59°C, 30sec	**NM005324**
	**rev** 5’-GAA ACC TCA GGT CGG TTT TG-3’			

The amplification program was as follows: 95°C/15min, followed by 47 cycles of denaturation at 94°C/15sec, annealing Ta* at 25sec or 30sec, extension at 72°C/15sec. Melting curves for each specific primer were monitored between 60°C–90°C, at the end of amplification, before a further extension at 75°C/5min. In bold, referring gene details;

*Ta, annealing temperature; bp, base-pairs of amplicons.

### ELISA

To evaluate NGF in the culture media, a two site NGF-ELISA (0.5pg/mL sensitivity and no cross-reactivity) was carried out in Maxisorp NUNC 96 well ELISA plates precoated with mouse anti-NGF antibodies (0.4μg/mL; MAB256, R&D) and incubated with standards (0.15pg/mL to 1ng/mL β-NGF; Alomone) or prediluted samples (1:3). The following steps included as follows: the biotinylated polyclonal anti-NGF antibodies (0.15μg/mL; BAF256, R&D), the streptavidin solution (Biosource International, Camarillo, CA) and the TMB substrate (Biosource). Optical density was detected by an ELISA plate reader (λ_450–550_; Sunrise; Tecan Systems, Inc., San Jose, CA) and calculations were carried out according to the protein normalization (A280 Nanodrop) [16]. The biological activity of NGF released by OCP-FBs was verified separately by using a PC12 bioassay (see [18]).

TGFβ1 and IL4 were measured in the conditioned media by commercially available ELISA kits, according to the manufacturers’ instructions (Biosource).

### Western Blotting

Total proteins were extracted from single-cells lysed in modified RIPA buffer [50 mM Tris-HCl (pH 7.7), 150mM NaCl, 1%Triton X-100 and 0.1% SDS] freshly supplemented with phosphatase/protease inhibitors (Pierce). Equivalent amounts of protein (30μg) were subjected to 7–15% SDS-PAGE electrophoresis (Miniprotean3; Bio-Rad, Hercules, CA) and resolved proteins were transferred to Hy-bond membranes (semi-dry blotting apparatus; Bio-Rad). The membranes were washed in 0.05% Tween 20 in PBS (TW-PBS), blocked in 5% non-fat dry milk-PBS and probed with trkA^NGFR^ (1/700), p75^NTR^(1/500), αSMA (1/500) and GAPDH (1/1000) primary antibodies, followed by secondary POD-conjugated specie-specific antibodies (1/10000; Jackson Immuneresearch). Developing was performed according to the ECL technique (SuperSignal West Pico Trial; Pierce, Rockford, IL). Bands were acquired/analysed in a Kodak Image station equipped with a 1D Image analysis software (110F; Kodak, Tokyo, Japan).

### Statistical Analysis

All experimental procedures were conducted in triplicate, each one repeated three times. Both molecular and biochemical data (means±SD) were analysed for significant differences (p < .05), by the ANOVA-Tukey-Kramer coupled post-hoc analysis (StatView II for PC; Abacus Concepts. Inc., Barkley, CA). REST-ANOVA analysis followed by Tukey Kramer posthoc comparisons was used to validate molecular data.

## Results

**Fig 1:**
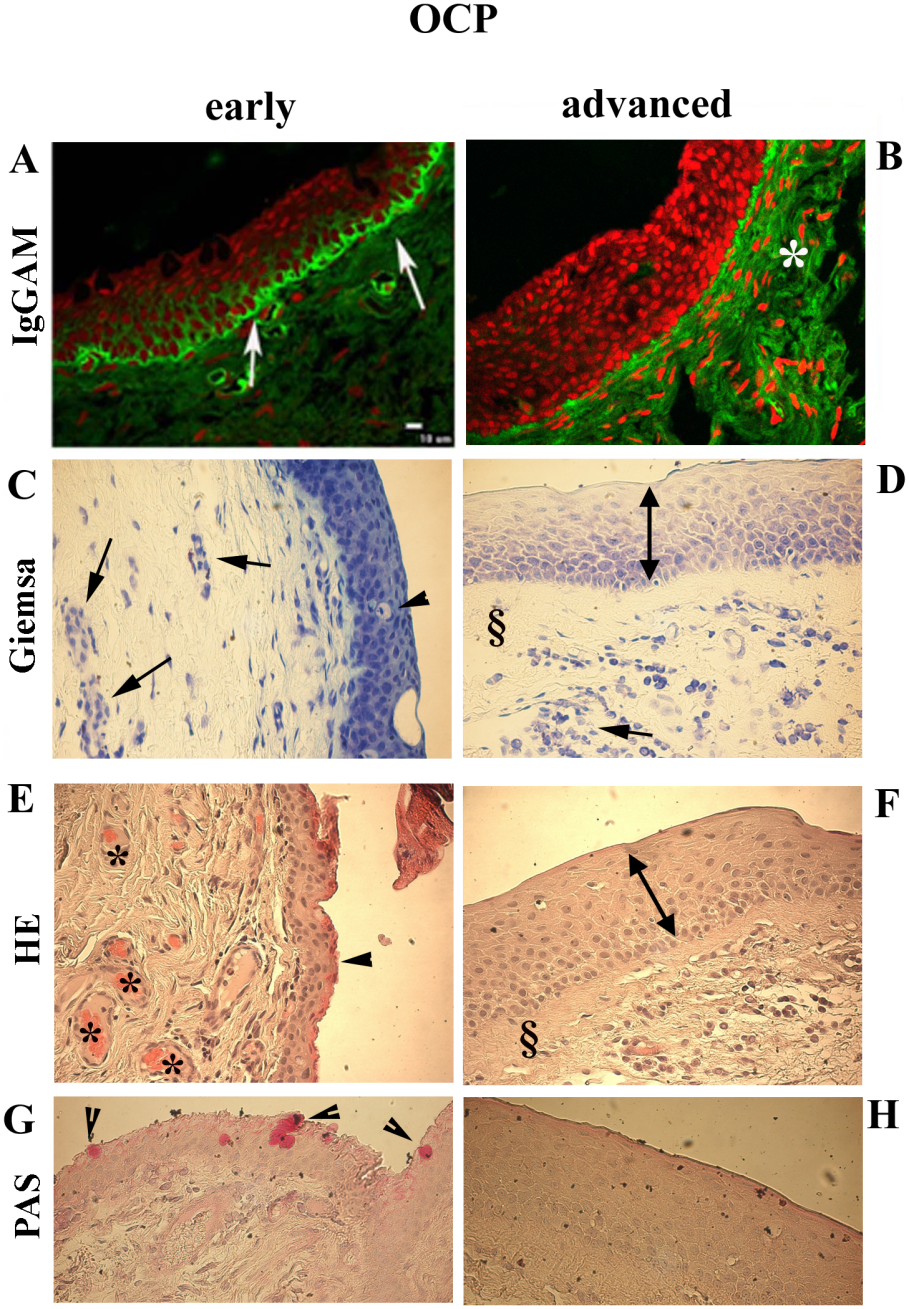
Histological characterization of OCP conjunctivas. Representative confocal (A-B) and light microscopy (C-H) images, including Giemsa (CD), HE (EF) and PAS (GH) stainings, from dewaxed conjunctival sections. A-B. Linear fluorescent-coupled IgGAM reactivity in the Basement Membrane Zone of *early* (white arrows; A) and *advanced* (B; the white asterisk indicates the stromal immunoreactivity) OCP sections. Fluorescent linear immunoreactivity is absent in normal sections. Depletion of goblet cells (pointed by black arrowheads) in *early* (C,E,G) with respect to the complete absence in *advanced* OCP (D,F,H); the presence of a squamous metaplasia (↔) particularly in *advanced* OCP (D,F); vessel ectasia (*) more prominent in *early* (E) than *advanced* (F); edema with mild infiltrates (arrows) in *early* (C) and to a less extend in *advanced* (D) OCP, particularly plasmacells and some granulocytes; and finally superficial homogenization of connective tissue (§), particularly evident in *advanced* OCP (D,F). Magnifications: x400.

Only conjunctival biopsies from patients’ with confirmed ophthalmic and microscopic diagnosis of OCP were included in this study. Immunofluorescent staining revealed the presence of a clear linear fluorescence alongside the BMZ, representative of autoantibodies deposition in both *early* (A) and *advanced* (B) OCP sections (Fig 1). No significant difference in IgGAM immunoreactivity was observed between *early* and *advanced* OCP, except for an increasing fluorescent signal in the epithelium and stroma (asterisk; Fig 1B). Contiguous to those IgGAM staining, basal histology was also performed by using Giemsa (Fig 1C and 1D), HE (Fig 1E and 1F) and PAS (Fig 1G and 1H). A depletion of goblet cells was observed in *early* OCP (arrowheads) while the absence of goblet cells was detected in *advanced* OCP, as pointed in Giemsa, HE and particularly PAS panels (Fig 1C–1H). The presence of squamous metaplasia (pluristratified non-keratinised epithelial with absence of goblet cells and homogenization of connective tissue) was particularly evident in *advanced* OCP (see § in Fig 1D and 1F). A marked vessel ectasia was particularly evident in *early* OCP (see asteriks in Fig 1E). Edema and mild infiltrates with prevalence of plasmacells (arrows) were observed in *early* with respect to *advanced* OCP (Fig 1C).

### Characterization of activated FBs

**Fig 2:**
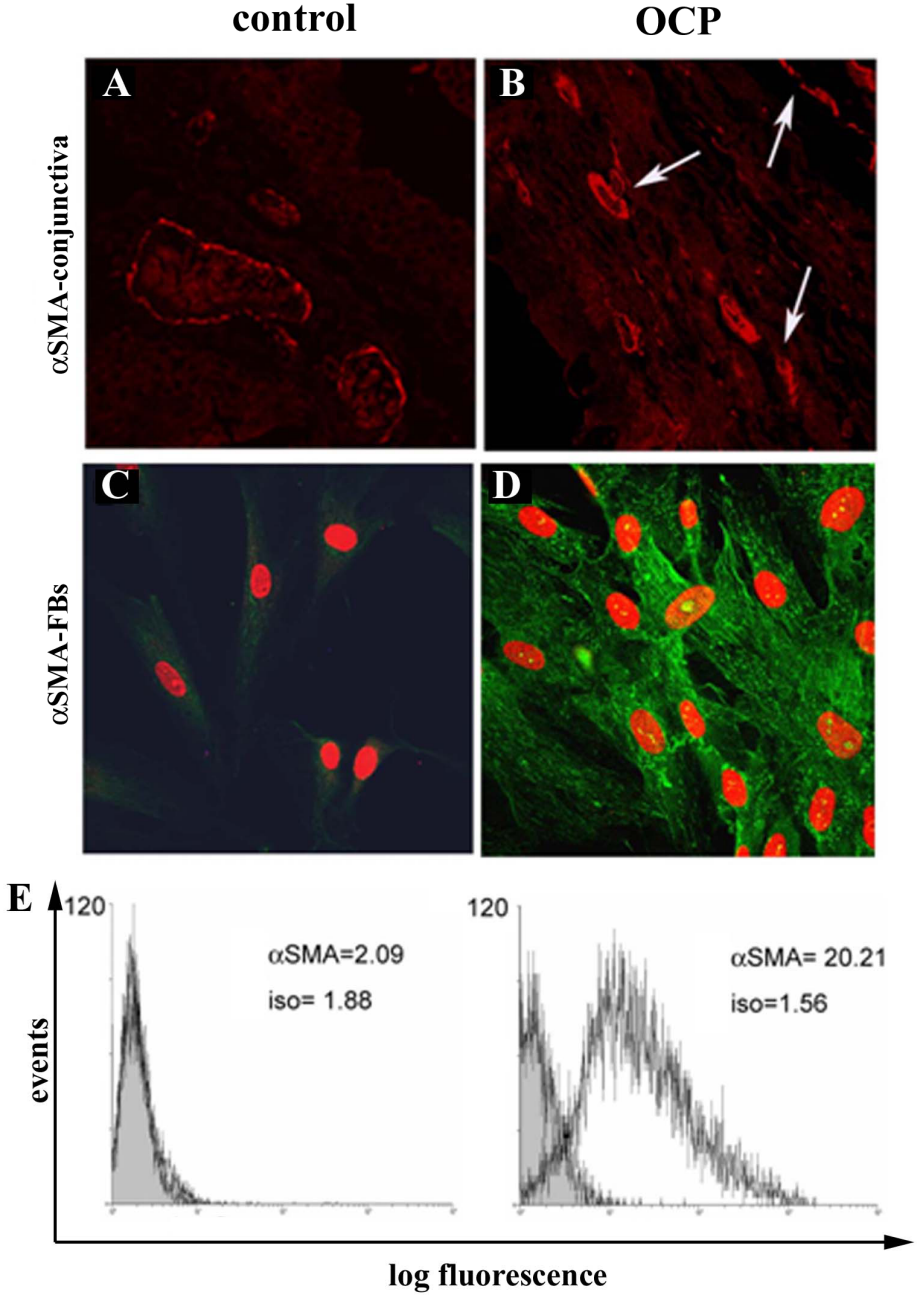
aSMA expression in both OCP conjunctiva and derived FBs. A-D. Confocal analysis for α-SMA in both control (A) and OCP (B) conjunctival biopsies, as well as in control (C) and OCP (D) FB. Arrows point to specific α-SMA immunoreactivity in OCP tissue (B; red staining; see M&M for details). In monolayers, nuclei were counterstained with propidium iodide (C-D). [Magnifications: AB, x400; CD, x600] E-F. Flow cytometry analysis specific for α-SMA in control (E) and OCP (F) FBs. iso = fluorescence intensity related to isotypes.

Adjacent conjunctival sections and primary cultures of FBs were analysed for αSMA expression by confocal microscopy and flow cytometry (FCM) analysis. As shown in Fig 2, αSMA immunoreactivity in outgrowth of OCP-tissue (B) and outgrew FBs (D), as compared to control specimens (respectively A and C; p < .05). FCM analysis confirmed the higher αSMA protein expression in OCP-FBs (Fig 2E; p < .05). FCM results were corroborated at the molecular level (+14.00±4.80 expression ratio in OCP-FBs *vs*. controls; p < .05).

### Expression of NGF and trkA^NGFR^/p75^NTR^

**Fig 3:**
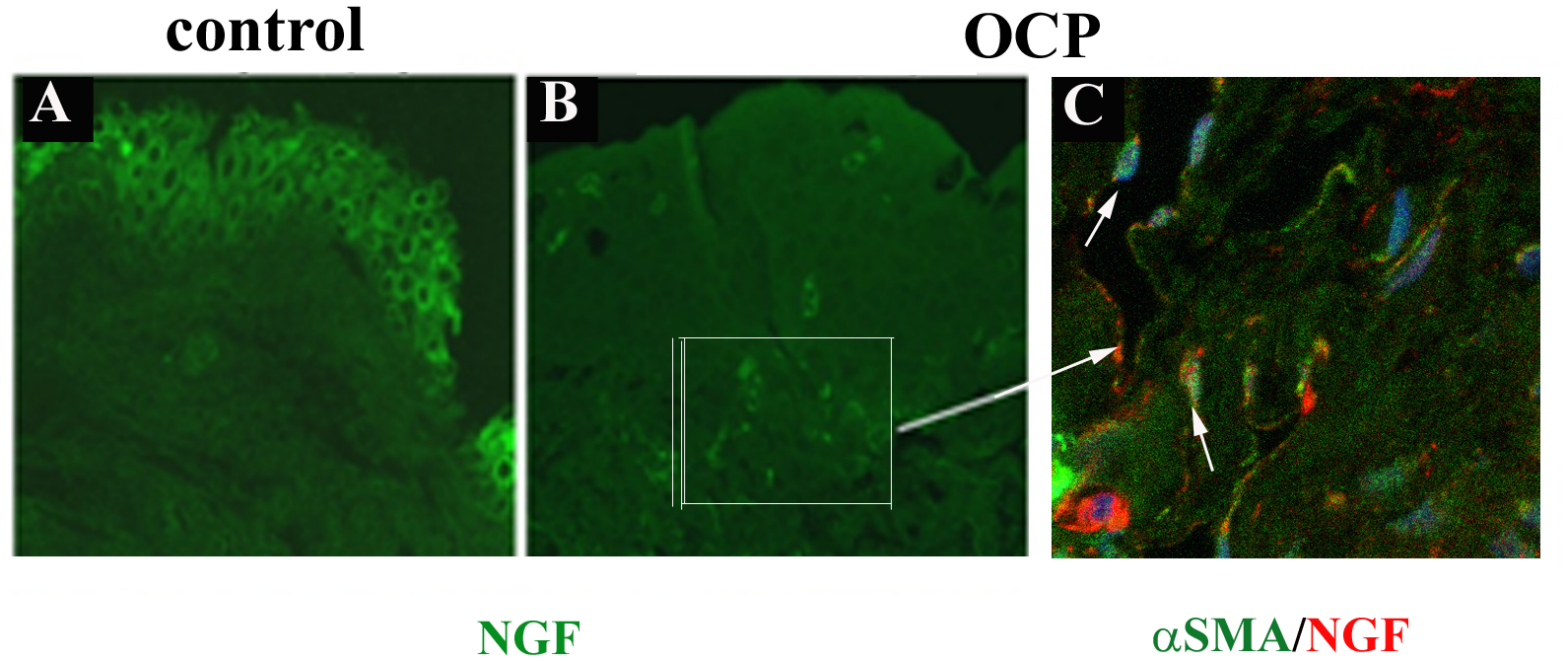
NGF immunoreactivity in OCP conjunctiva and FBs. Confocal analysis for NGF (green) in control (A) and OCP (B,C) conjunctiva. C. Immunofluorescence for NGF (red) and αSMA (green) expression in the conjunctival stroma. Note the presence of intracytoplasm and perinuclear staining. Nuclei were counterstained with toto3. Magnifications: AB, x400; C, x600 (oil immersion).

OCP conjunctiva and OCP-FBs were examined for NGF-trkA^NGFR^/p75^NTR^ expression. As observed by confocal analysis, NGF expression was decreased in the epithelium and specifically increased in OCP stroma, as compared to controls **(** Fig 3A and 3B; p < .05). Indeed, confocal analysis showed higher cytoplasmic and perinuclear NGF immunoreactivity in outgrew OCP-FBs, supporting those data observed in OCP stroma (see arrows; Fig 3C). In addition, the conditioned media from OCP-FBs showed higher NGF levels than those of control counterparts (420.00±90.00 *vs*. 220.00±64.00 pg/mL; p < .05), as detected by NGF ELISA. In line, NGFmRNA was increased in these OCP-FBs, as compared to their control FBs (+5.00±1.30 expression ratio; p < .05). No significant difference was quantified between *early* and *advanced* specimens.

**Fig 4:**
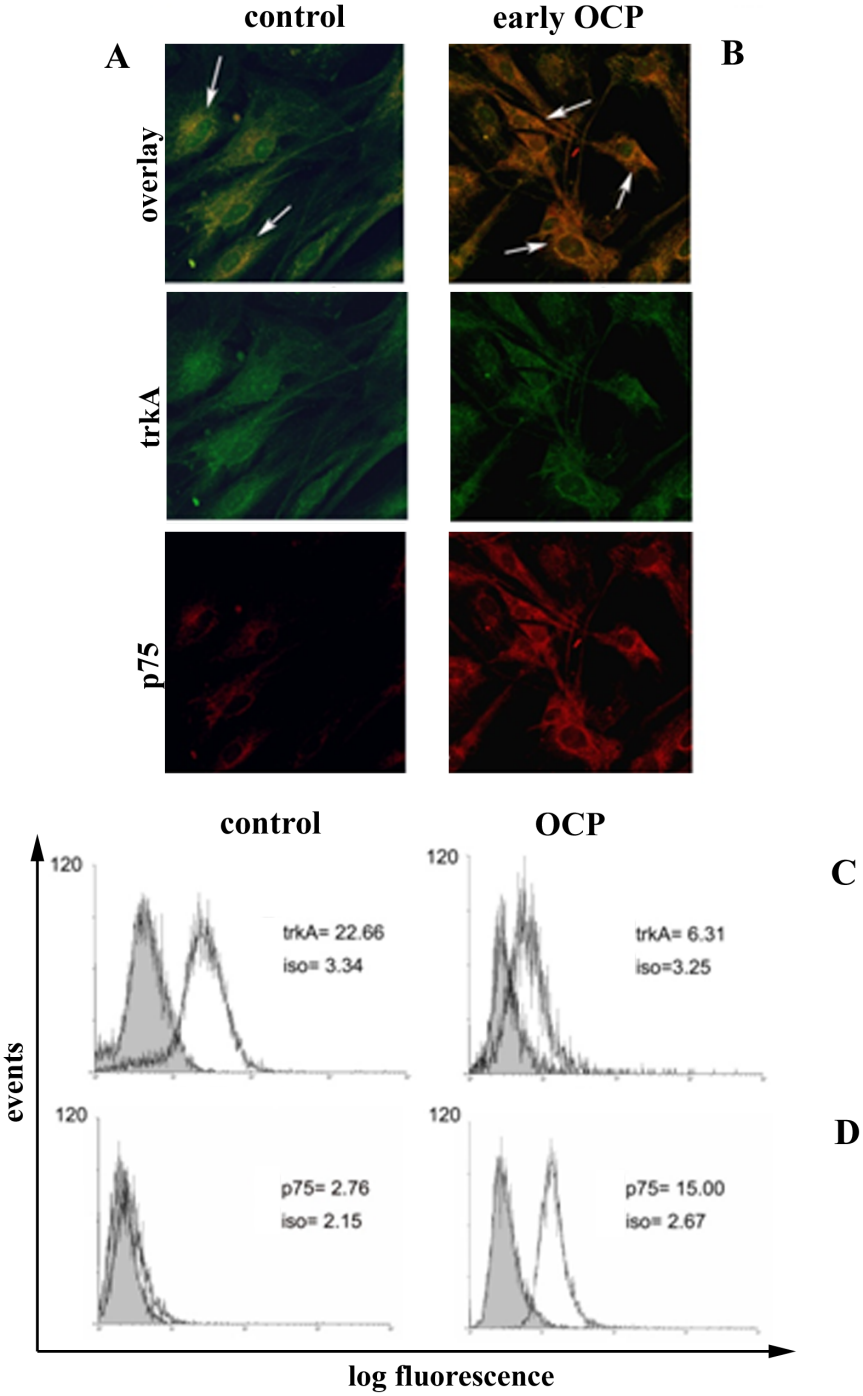
trkA^NGFR^ and p75^NTR^ in OCP conjunctiva and FBs. AB. Confocal analysis of control (A) and OCP (B) FBs double (overlays; x400) and single stained for trkA^NGFR^ and p75^NTR^ (see below). Relevant single immunoreactions are shown below and cross-reactivity of trkA^NGFR^ and p75^NTR^ are marked with white arrows (overlays). CD: Flow cytometry analysis of control (left) and OCP (right) FBs showing expression of trkA^NGFR^ (C) and p75^NTR^ (D). Related isotype fluorescence intensity data are shown (iso).

Confocal analysis showed a high p75^NTR^ expression associated with a weak trkA^NGFR^ expression in OCP-FBs (Fig 4B). By contrary, control FBs showed a huge trkA^NGFR^ expression with respect to p75^NTR^ slightly expressed (Fig 4A). To support, the related FCM analysis for trkA^NGFR^ and p75^NTR^ are provided in Fig 4C and 4D respectively.

### αSMA and p75^NTR^co-expression in OCP-derived activated FBs

**Fig 5:**
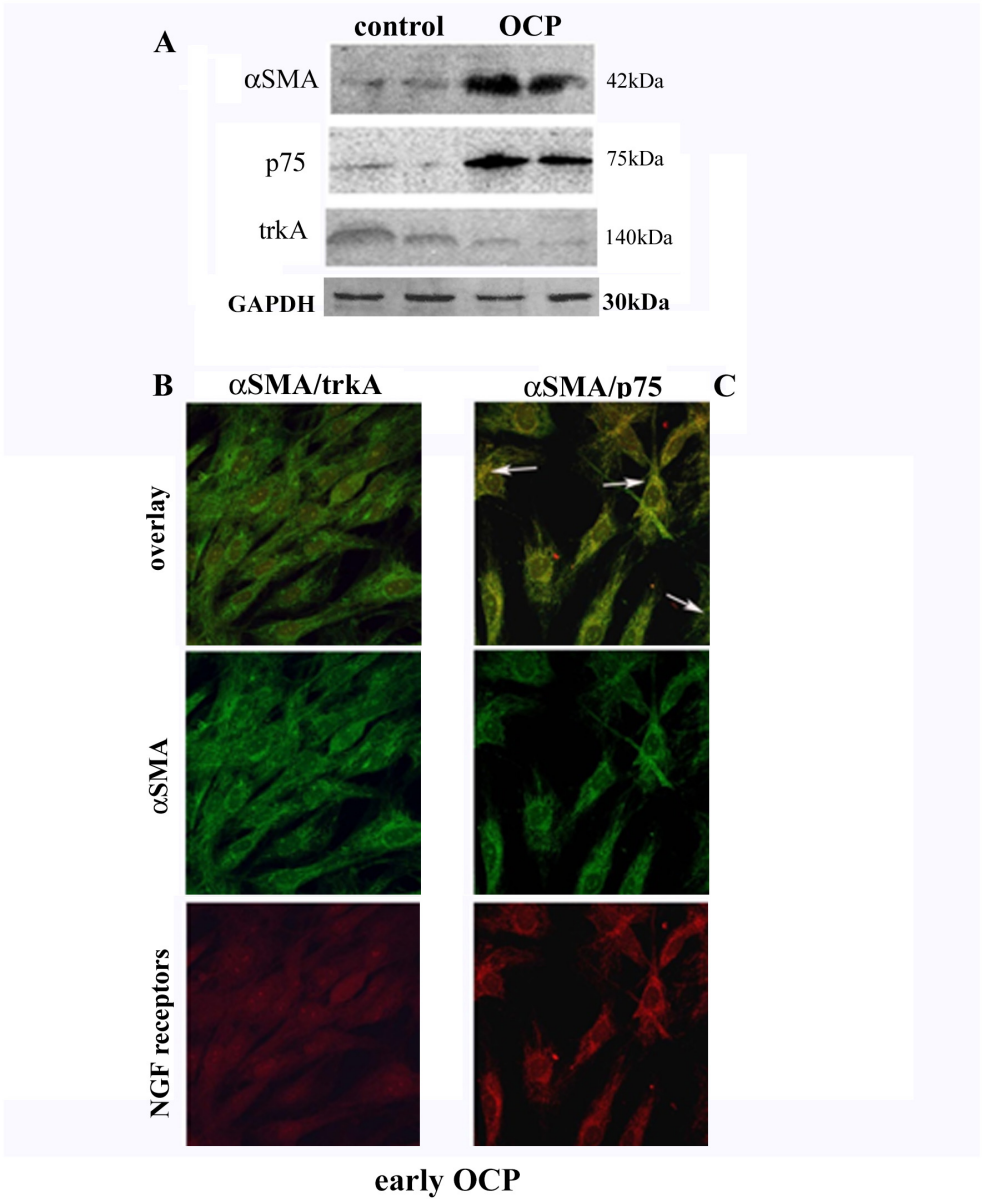
αSMA and trkA^NGFR^/p75^NTR^ expression in OCP-FBs. A. Representative Western blot analysis specific for αSMA, p75^NTR^ and trkA^NGFR^ proteins in control (left) and OCP (right) FBs (n = 2/each group). Normalization was checked by GAPDH reprobing on the same gels. BC. Confocal analysis for α-SMA/trkA^NGFR^ (B) and α-SMA/p75^NTR^(C) in OCP-FBs (overlays, x600). Respective single immunoreactions are shown below. p75^NTR^ and α-SMA cross-reactivity is highlighted with white arrows (C).

The higher αSMA and p75^NTR^ and the lower trkA^NGFR^ protein expressions in OCP-FBs were also detected by WB analysis, as compared to control FBs (Fig 5A; p<0.05). As shown in Fig 5B, αSMA protein co-localized with p75^NTR^ but not with trkA^NGFR^, as detected by confocal analysis. This would suggest the existence of a specific trkA^NGFR^/p75^NTR^ expression ratio during the progress of fibrosis. Particularly, basal histology showed a strong infiltration of inflammatory cells (arrows pointing to plasmacells in Fig 1C), the presence of connective tissue homogenization (asterisk in 1D and 1F) in the underlying stroma (§) and a significant decrease (absence) of goblet cells in the epithelium of *advanced* OCP sections (see arrowheads pointing residual goblet cells in *early* OCP) (Fig 1C and 1E). Activated fibroblast were also visible in *early* OCP (Fig 1C). No significant difference in αSMA, p75^NTR^ and trkA^NGFR^ transcripts was detected in *early* OCP-FBs compared to controls (respectively +1.05±05, +1.30±0.50 and +2.00±1.00 expression ratio; p>.05). In *advanced* OCP*-*FBs, αSMA and p75^NTR^ transcripts were significantly increased (+9.00±0.40 and +5.00±0.50 expression ratio; p < .001) while trkA^NGFR^ transcript was significantly decreased (-4.00±1.00 expression ratio; p < .05), as compared to controls. By FCM analysis, αSMA protein was increased in *early* OCP-FBs (2.8±0.58 *vs*. 0.22±0.06 MFI, early *vs*. controls; p>.05) and significantly increased in *advanced* OCP-FBs (27.05±3.6 *vs*. 0.22±0.06 MFI, advanced *vs*. controls; p < .001). Indeed, p75^NTR^ protein was increased in both *early* (15.52±5.97 *vs*. 1.44±0.6 MFI, early *vs*. controls; p < .05) and *advanced* (17.0±3.0 *vs*.1.44±0.6 MFI, advanced *vs*. controls; p < .01) OCP-FBs. Finally, trkA^NGFR^ protein expression in *early* OCP-FBs was comparable to those of control-FBs while it decreased in *advanced* OCP-FBs (2.41±1.27 *vs*. 15.5±1.00 MFI, *advanced vs*. controls; p < .01).

### NGF modulation of αSMA and trkA^NGFR^/p75^NTR^expression in OCP-FBs

**Fig 6:**
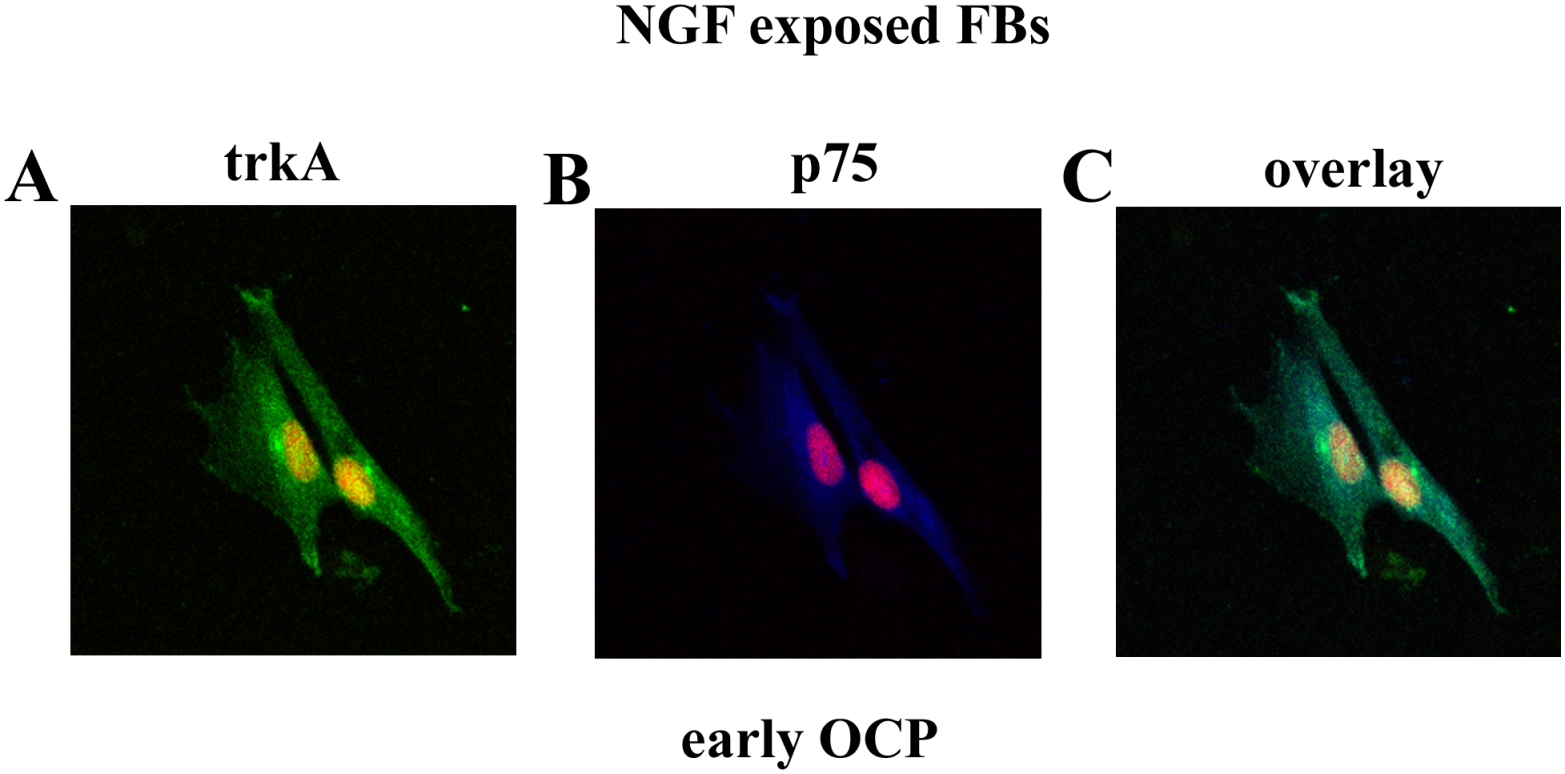
trkA^NGFR^/p75^NTR^ expression in NGF exposed early OCP-FBs. Confocal images showing the trkA^NGFR^ (FC/green, A) and p75^NTR^ (Cy5/blue, B) immunoreactivity in early OCP-FBs exposed to NGF over 24hrs (overlays, C). Nuclei counterstained with propidium iodide are shown in all panels. The cytoplasmic and perinuclear distribution of both receptors is clearly visible. Magnifications: A-C, x400.

NGF effect on αSMA and trkA^NGFR^/p75^NTR^ modulation was thereafter investigated by exposure to 10ng/mL NGF over 24hrs. A preliminary dose response study was carried out on OCP-FBs (0–100 ng/mL NGF over 24hrs), highlighting the 10 ng/mL NGF dosage. In *early* OCP-FBs, decreased αSMA and p75^NTR^ (respectively 0.83±0.19 *vs*. 2.8±0.58 MFI and 1.08±0.56 *vs*. 15.52±5.97 MFI; p < .05) as well as unchanged trkA^NGFR^ (20.33±8.52 *vs*. 20.93±4.87 MFI; p>.05) protein expressions were observed upon NGF exposure. In *advanced* OCP-FBs, no significant changes of αSMA and p75^NTR^ (respectively 17.60±1.15 *vs*. 17.00±6.12 and 31±4.77 *vs*. 26.83±3.84 MFI; p>.05) as well as trkA^NGFR^ (2.93±0.83 *vs*. 2.41±1.27 MFI; p>.05) protein expressions were detected upon NGF exposure. With respect to trkA^NGFR^/p75^NTR^ expression, FCM analysis showed that 94.91% *early* OCP-FBs were trkA^NGFR^ positive, with 57.24% co-expressing p75^NTR^. Upon NGF exposure, 96.88% *early* OCP-FBs were still trkA^NGFR^ positive, with 24.12% co-expressing p75^NTR^ and 72.76% expressing only trkA^NGFR^. The statistical analysis showed that a decrease of 57% in trkA^NGFR^/p75^NTR^ co-expressing cells occurred in association with a shift to trkA^NGFR^ expressing cells. The trkA^NGFR^/p75^NTR^ immunoreactivity in NGF exposed early OCP-FBs is shown (Fig 6).

### NGF modulation of OCP-activated FBs derived TGFβ1 and IL4 cytokines

Last, changes in TGFβ1 and IL4 profibrogenic factor release were also detected in the conditioned media from baseline and NGF treated OCP-FBs. TGFβ1 and IL4 levels in the conditioned media from *early* OCP-FBs were respectively 8-times (101.00±30.00 *vs*. 12.00±2.10 pg/mL TGFβ1, p < .05) and 6-times (308.00±7.00 *vs*. 55.00±40.00 pg/mL IL4; p < .001) higher as compared to control counterparts. Conditioned media from *advanced* OCP-FBs did not show difference in both TGFβ1 and IL4 levels, as compared to controls. Upon 10ng/mL NGF exposure, IL4 protein decreased in the conditioned media from *early* (26.00±10.00 *vs*. 101.00±30.00 pg/mL IL4; p < .05) and *advanced* (1.80±0.30 vs. 23.00±4.80 pg/mL IL4; p < .05) OCP-FBs. By contrary, TGFβ1 levels decreased only in the conditioned media from *early* OCP-FBs (63.00±40.00 vs. 308.00±7.00 pg/mL TGFβ1; p < .05).

## Discussion

Increasing data indicate that the chronic inflammatory process occurring in OCP conjunctiva leads to FB activation and survival, with overt collagen deposition and excessive matrix deposition [3]. As a product of different structural/immune cells and FBs/myoFBs, both cytokines and growth factors actively contribute to subepithelial fibrosis and conjunctival scarring [25]. To date, different proinflammatory and profibrogenic factors have been investigated by different groups, which have focused their attention especially on receptor signalling [26]. While in a previous study we have described the trkA^NGFR^ and NGF expression respectively in OCP conjunctivas and tears, herein we hypothesize a possible NGF role in the modulation of cultured OCP-FBs [8,9].

First of all, αSMA expression was detected in OCP conjunctiva and confirmed in primary cultures of FBs obtained from OCP explants. αSMA (α-Smooth Muscle Actin) represents the most reliable phenotypic marker for the majority of fibrotic states. Our findings indicate the presence of activated FBs inside inflamed/fibrotic OCP conjunctiva [7,27,28]. If these activated FBs are αSMA-expressing myofibroblast (myoFBs) remains to be clarified since in previous studies the possible differentiation of OCP-FBs into myoFBs was not reported [29].

An increase in the stroma and a significant decrease in the epithelium were detected for NGF in OCP conjunctival biopsies (n = 7), as compared to control ones. To the best of our knowledge, this data has not been previously described and it is supported by our recent findings showing an increased NGF content in OCP tear fluids [8,9]. The observation of NGF-expressing OCP-FBs might suggest that the NGF increase in OCP stroma as well as the increased NGF levels in OCP tears might be partially due to local activated-FBs. On the other side, the decreased NGF immunoreactivity in OCP epithelium is actually missing of explanation and not investigated/discussed in this study.

As product of stromal inflammation, it is reasonable to hypothesize that NGF might contribute to tissue remodeling by influencing the FB phenotype, as observed in previous studies conducted on other cell types [14,17,18,20]. With respect to the activated FB phenotype, NGF effects might cover either cell survival and/or soluble mediator release [14,17]. According to literature, NGF activity is driven by trkA^NGFR^ and p75^NTR^ receptors, which mediate NGF signal alone or in cooperation [30–35]. As a new finding, NGF, trkA^NGFR^/p75^NTR^ and αSMA (co)expressions were detected in primary cell cultures alongside with sub-cultured OCP-FBs. Of interest, sub-cultured OCP-FBs showed the ability to retain FB phenotype upon few passages and were therefore suitable for stimulation studies. Interestingly, trkA^NGFR^/p75^NGF^ expression was found strictly dependent to the *early/advanced* grouping of disease as well as αSMA phenotype correlated to p75^NTR^ and paralleled the severity of fibrosis. Particularly, FBs from *advanced* OCP showed higher αSMA and p75^NTR^ together with lower trkA^NGFR^, as compared to *early* and control counterparts. This expression would imply a close association of p75^NTR^ with OCP-FB phenotype, and highlight a possible modulation of myoFB apoptosis, as observed in other systems [20,35]. To date, the role of trkA^NGFR^ and p75^NTR^ in tissue remodeling remains controversial. As documented, both trkA^NGFR^ and p75^NTR^ can mediate either survival or apoptosis, depending on their surface receptor (co)expression and microenvironment [33,36–38]. In early healing process, high levels of trkA^NGFR^ might drive both migration and differentiation (as initial matrix remodelling) while in late healing process the trkA^NGFR^ down-regulation might allow p75^NTR^ to mediate other biological activities, alone or eventually in cooperation with trkA^NGFR^ [33,39–41]. As described, fibrotic tissues appear characterized by low trkA^NGFR^ and high p75^NTR^ expression [17,18,20]. In this study, a higher trkA^NGFR^/p75^NTR^ ratio (the outcome of a trkA^NGFR^ over-expression) was observed in *early* OCP-FBs while lower trkA^NGFR^/p75^NTR^ ratio (the outcome of an increased p75^NTR^ expression) was detected in *advanced* OCP-FBs, according to the clinical and histological features (infiltrates and remodelling features) [2,22,42]. To support our findings, the lower trkA^NGFR^/p75^NTR^ ratio expression in *advanced* OCP-FBs (the outcome of an increased p75^NTR^ expression) has been also reported in other fibrotic conditions, either in vitro/ex vivo [7,17,18,28,43]. A down-regulation of both αSMA and p75^NTR^ expression was observed in NGF-exposed *early* OCP-FBs, while no effect was detected in NGF-exposed *advanced* counterpart. The observation that NGF modulated trkA^NGFR^/p75^NTR^ ratio expression preferentially in early OCP-FBs would suggest that a potential control of activated FBs might be possible in early OCP showing a mild-moderate clinical facet, opening to potential NGF therapeutic applications. As shown, activated FBs disappear alongside “proper repair process” while αSMA-expressing activated FBs survive in pathological remodelling [7,28]. This process might be highly regulated by growth factors and cytokines, including NGF, all known to be increased in OCP tissues and tears [5,6,8,9]. Therefore, a possible cross-talk between NGF and other profibrogenic factors cannot be excluded. In line, TGFβ1 and IL4 were extensively investigated in fibrosis and are widely reported to contribute selectively to tissue remodelling and overt fibrosis in different disorders via an extensive sustaining of myoFBs [2,6,28,44]. Therefore, we wonder whether NGF might influence TGFβ1 and IL4 release from sub-cultures of OCP-FBs. The biochemical analysis highlighted a significant decrease of TGFβ1 and IL4 in the conditioned media from NGF-exposed *early* OCP-FBs, while only a decrease of IL4 was monitored in *advanced* counterparts. This selective effect holds up the potential NGF involvement in OCP remodelling, through a modulation of inflammatory/fibrogenic soluble factors, at least in *early* stage of disease.

Overall, OCP is a chronic inflammatory disease that slowly evolves in severe conjunctival scarring and visual impairments [1,2,45]. Most of the current OCP therapies target the suppression of inflammation, as counteracting the recurrent inflammation represents the main way to reduce progressive remodelling [46–48]. The findings of this *in vitro* study suggest a possible NGF effect on early OCP-FBs having a low trkA^NGFR^/p75^NTR^ ratio, highlighting the possible NGF effect in the modulation of FB activity during the *early* stages of disease. Since the topical NGF application has been suggested as a therapeutic tool in some ocular surface disorders [11,49], these findings encourage further studies to understand the underlying NGF mechanism in OCP conjunctiva in order to develop alternative strategies to counteract fibrosis.

## References

[1] Ahmed M, Zein G, Khawaja F, Foster CS. Ocular cicatricial pemphigoid: pathogenesis, diagnosis and treatment. Prog Retin Eye Res 2004; 23: 579–592. 15388075 10.1016/j.preteyeres.2004.05.005

[2] Foster CS, Sainz De La Maza M. Ocular cicatricial pemphigoid review. Curr Opin Allergy Clin Immunol 2004; 4: 435–439. 15349045 10.1097/00130832-200410000-00017

[3] Razzaque MS, Foster CS, Ahmed AR. Tissue and molecular events in human conjunctival scarring in ocular cicatricial pemphigoid. Histol Histopathol 2001; 16: 1203–1212. 11642740 10.14670/HH-16.1203

[4] Kirzhner M, Jakobiec FA. Ocular cicatricial pemphigoid: a review of clinical features, immunopathology, differential diagnosis, and current management. Semin Ophthalmol 2011; 26: 270–277. 10.3109/08820538.2011.588660 21958173

[5] Razzaque MS, Foster CS, Ahmed AR. Role of connective tissue growth factor in the pathogenesis of conjunctival scarring in ocular cicatricial pemphigoid. Invest Ophthalmol Vis Sci 2003; 44: 1998–2003. 12714635 10.1167/iovs.02-0967

[6] Razzaque MS, Ahmed BS, Foster CS, Ahmed AR. Effects of IL-4 on Conjunctival Fibroblasts: Possible Role in Ocular Cicatricial Pemphigoid. Invest Ophthalmol Vis Sci 2003; 44: 3417–3423. 12882790 10.1167/iovs.02-1084

[7] Desmouliere A, Darby IA, Gabbiani G. Normal and pathologic soft tissue remodeling: role of the myofibroblast, with special emphasis on liver and kidney fibrosis. Lab Invest 2003; 83: 1689–1707. 14691287 10.1097/01.lab.0000101911.53973.90

[8] Lambiase A, Bonini S, Micera A, Rama P, Bonini S, Aloe L. Expression of nerve growth factor receptors on the ocular surface in healthy subjects and during manifestation of inflammatory diseases. Invest Ophthalmol Vis Sci 1998; 39: 1272–1275. 9620090

[9] Lambiase A, Micera A, Sacchetti M, Cortes M, Mantelli F, Bonini S. Alterations of tear neuromediators in dry eye disease. Arch Ophthalmol 2011; 129: 981–986. 10.1001/archophthalmol.2011.200 21825181

[10] Levi-Montalcini R. The saga of the nerve growth factor. Neuroreport 1998; 9: 71–83. 9858356

[11] Aloe L, Micera A. Nerve Growth Factor: Basic finding and clinical trials. Biomedical Reviews 1999; 10: 3–14.

[12] Bonini S, Lambiase A, Bonini S, Levi-Schaffer F, Aloe L. Nerve growth factor: an important molecule in allergic inflammation and tissue remodelling. Int Arch Allergy Immunol 1999; 118: 159–162. 10224366 10.1159/000024055

[13] Chao MV. Neurotrophins and their receptors: a convergence point for many signalling pathways. Nat Rev Neurosci 2003; 4: 299–309. 12671646 10.1038/nrn1078

[14] Micera A, Lambiase A, Aloe L, Bonini S, Levi-Schaffer F, Bonini S. Nerve growth factor involvement in the visual system: implications in allergic and neurodegenerative diseases. Cytokine Growth Factor Rev 2004; 15: 411–417. 15561599 10.1016/j.cytogfr.2004.09.003

[15] Micera A, Lambiase A, Stampachiacchiere B, Sgrulletta R, Normando EM, Bonini S et al Nerve growth factor has a modulatory role on human primary fibroblast cultures derived from vernal keratoconjunctivitis-affected conjunctiva. Mol Vision 2007; 13: 981–987. PMC2774460 17653039

[16] Hempstead BL. Dissecting the diverse actions of pro- and mature neurotrophins. Curr Alzheimer Res 2006; 3: 19–24. 16472198 10.2174/156720506775697061

[17] Micera A, Lambiase A, Stampachiacchiere B, Bonini S, Bonini S, Levi-Schaffer F. Nerve growth factor and tissue repair remodeling: trkA(NGFR) and p75(NTR), two receptors one fate. Cytokine Growth Factor Rev 2007; 18: 245–256. 17531524 10.1016/j.cytogfr.2007.04.004

[18] Micera A, Vigneti E, Pickholtz D, Reich R, Pappo O, Bonini S, et al Nerve growth factor displays stimulatory effects on human skin and lung fibroblasts, demonstrating a direct role for this factor in tissue repair. Proc Natl Acad Sci USA 2001; 98: 6162–6167. 11344264 10.1073/pnas.101130898 PMC33439

[19] Kendall TJ, Hennedige S, Aucott RL, Hartland SN, Vernon MA, Benyon RC, et al p75 Neurotrophin receptor signaling regulates hepatic myofibroblast proliferation and apoptosis in recovery from rodent liver fibrosis. Hepatology 2009; 49: 901–910. 10.1002/hep.22701 19072833

[20] Trim N, Morgan S, Evans M, Issa R, Fine D, Afford S, et al Hepatic stellate cells express the low affinity nerve growth factor receptor p75 and undergo apoptosis in response to nerve growth factor stimulation. Am J Pathol 2000; 156: 1235–1243. 10751349 10.1016/S0002-9440(10)64994-2 PMC1876895

[21] Lee R, Kermani P, Teng KK, Hempstead BL. Regulation of cell survival by secreted proneurotrophins. Science 2001; 294: 1945–1948. 11729324 10.1126/science.1065057

[22] Mondino BJ, Brown SI. Ocular cicatricial pemphigoid. Ophthalmology 1981; 88: 95–100. 7015218 10.1016/s0161-6420(81)35069-6

[23] Williams GP, Radford C, Nightingale P, Dart JK, Rauz S. Evaluation of early and late presentation of patients with ocular mucous membrane pemphigoid to two major tertiary referral hospitals in the United Kingdom. Eye 2011; 25: 1207–1218. 10.1038/eye.2011.175 21799523 PMC3173873

[24] Pfaffl MW, Horgan GW, Dempfle L. Relative expression software tool (REST) for group-wise comparison and statistical analysis of relative expression results in real-time PCR. Nucleic Acids Res 2002; 30: e36 11972351 10.1093/nar/30.9.e36 PMC113859

[25] Jelaska A, Strehlow D, Korn JH. Fibroblast heterogeneity in physiological conditions and fibrotic disease. Springer Semin Immunopathol 1999; 21: 385–395. 10945032

[26] Wynn TA, Ramalingam TR. Mechanisms of fibrosis: therapeutic translation for fibrotic disease. Nature Medicine 2012; 18: 1028–1040. 10.1038/nm.2807 22772564 PMC3405917

[27] Tomasek JJ, Gabbiani G, Hinz B, Chaponnier C, Brown RA. Myofibroblasts and mechano-regulation of connective tissue remodelling. Nat Rev Mol Cell Biol 2002; 3: 349–363. 11988769 10.1038/nrm809

[28] Hinz B. Formation and function of the myofibroblast during tissue repair. J Invest Dermatol 2007; 127: 526–537. 17299435 10.1038/sj.jid.5700613

[29] Saw VP, Schmidt E, Offiah I, Galatowicz G, Zillikens D, Dart JK, et al Profibrotic phenotype of conjunctival fibroblasts from mucous membrane pemphigoid. Am J Pathol 2011; 178: 187–197. 10.1016/j.ajpath.2010.11.013 21224056 PMC3069913

[30] Sofroniew MV, Howe CL, Mobley WC. Nerve growth factor signaling, neuroprotection, and neural repair. Annu Rev Neurosci 2001; 24: 1217–1281. 11520933 10.1146/annurev.neuro.24.1.1217

[31] Mahadeo D, Kaplan L, Chao MV, Hempstead BL. High affinity nerve growth factor binding displays a faster rate of association than p140trk binding. Implications for multi-subunit polypeptide receptors. J Biol Chem 1994; 269: 6884–6891. 8120051

[32] Bibel M, Hoppe E, Barde YA. Biochemical and functional interactions between the neurotrophin receptors trk and p75NTR. EMBO J 1999; 18: 616–622. 9927421 10.1093/emboj/18.3.616 PMC1171154

[33] Zampieri N, Chao MV. Mechanisms of neurotrophin receptor signalling. Biochem Soc Trans 2006; 34: 607–611. 16856873 10.1042/BST0340607

[34] Nykjaer A, Willnow TE, Petersen CM. p75NTR-live or let die. Curr Opin Neurobiol 2005; 15: 49–57. 15721744 10.1016/j.conb.2005.01.004

[35] Passino MA, Adams RA, Sikorski SL, Akassoglou K. Regulation of hepatic stellate cell differentiation by the neurotrophin receptor p75NTR. Science 2007; 315: 1853–1856. 17395831 10.1126/science.1137603

[36] Matrone C, Marolda R, Ciafrè S, Ciotti MT, Mercanti D, Calissano P. Tyrosine kinase nerve growth factor receptor switches from prosurvival to proapoptotic activity via Abeta-mediated phosphorylation. Proc Natl Acad Sci U S A 2009; 106: 11358–11363. 10.1073/pnas.0904998106 19549834 PMC2699376

[37] Zhou Y, Lu TJ, Xiong ZQ. NGF-dependent retrograde signaling: survival versus death. Cell Res 2009; 19: 525–526. 10.1038/cr.2009.47 19421238

[38] Kraft AD, McPherson CA, Harry GJ. Heterogeneity of microglia and TNF signaling as determinants for neuronal death or survival. Neurotoxicology 2009; 30: 785–793. 10.1016/j.neuro.2009.07.001 19596372 PMC3329780

[39] Barker PA. p75NTR is positively promiscuous: novel partners and new insights. Neuron 2004; 42: 529–533. 15157416 10.1016/j.neuron.2004.04.001

[40] Teng KK, Hempstead BL. Neurotrophins and their receptors: signaling trios in complex biological systems. Cell Mol Life Sci 2004; 61: 35–48. 14704852 10.1007/s00018-003-3099-3 PMC11138791

[41] Schor NF. The p75 neurotrophin receptor in human development and disease. Prog Neurobiol 2005; 77: 201–214. 16297524 10.1016/j.pneurobio.2005.10.006

[42] Elder MJ, Bernauer W, Leonard J, Dart JK Progression of disease in ocular cicatricial pemphigoid. British Journal of Ophthalmology 1996; 80: 292–296. 8703876 10.1136/bjo.80.4.292 PMC505451

[43] Chen K, Wei Y, Sharp GC, Braley-Mullen H. Balance of proliferation and cell death between thyrocytes and myofibroblasts regulates thyroid fibrosis in granulomatous experimental autoimmune thyroiditis (G-EAT). J Leukoc Biol 2005; 77: 166–172. 15536125 10.1189/jlb.0904538

[44] Border WA, Noble NA. Mechanisms of disease: transforming growth factor (beta) in tissue fibrosis. N Engl J Med 1994; 331: 1286–1292. 7935686 10.1056/NEJM199411103311907

[45] Sgrulletta R, Lambiase A, Micera A, Bonini S. Corneal ulcer as an atypical presentation of ocular cicatricial pemphigoid. Eur J Ophthalmol 2007; 17: 121–123. 17294392 10.1177/112067210701700117

[46] Miserocchi E, Baltatzis S, Roque MR, Ahmed AR, Foster CS. The effect of treatment and its related side effects in patients with severe ocular cicatricial pemphigoid. Ophthalmology 2002; 109: 111–118. 11772589 10.1016/s0161-6420(01)00863-6

[47] Sacher C, Hunzelmann N. Cicatricial pemphigoid (mucous membrane pemphigoid): current and emerging therapeutic approaches. Am J Clin Dermatol 2005; 6: 93–103. 15799681 10.2165/00128071-200506020-00004

[48] Durrani K, Zakka FR, Ahmed M, Memon M, Siddique SS, Foster CS Systemic therapy with conventional and novel immunomodulatory agents for ocular inflammatory disease. Surv Ophthalmol 2011; 56: 474–510. 10.1016/j.survophthal.2011.05.003 22117884

[49] Lambiase A, Mantelli F, Sacchetti M, Rossi S, Aloe L, Bonini S. Clinical applications of NGF in ocular diseases. Arch Ital Biol 2011; 149: 283–292. 10.4449/aib.v149i2.1363 21702001

